# Intraoperative Ultrasound-Guided Selective Glossopharyngeal Nerve Block for Post-tonsillectomy Analgesia: A Case Report

**DOI:** 10.7759/cureus.56748

**Published:** 2024-03-22

**Authors:** Masataka Sekiguchi, Yuki Kojima, Akihiro Oue, Kazuya Hirabayashi

**Affiliations:** 1 Department of Otolaryngology-Head and Neck Surgery, Asahi General Hospital, Asahi, JPN; 2 Department of Anesthesiology, Asahi General Hospital, Asahi, JPN; 3 Department of Dental Anesthesiology, Asahi General Hospital, Asahi, JPN

**Keywords:** ultrasound nerve block, glossopharyngeal nerve, palatine tonsillectomy, anaesthetics, local analgesia

## Abstract

Tonsillectomy can lead to significant postoperative pain, which can impact the recovery process. Traditional analgesic approaches may entail risks due to medication use. Considering that the tonsils are innervated by the glossopharyngeal and maxillary nerves, implementing glossopharyngeal and maxillary nerve blocks can provide analgesia. Ultrasound guidance may improve its effectiveness and safety. A woman in her 30s with recurrent tonsillitis underwent tonsillectomy under general anesthesia. After induction, we performed an ultrasound-guided selective glossopharyngeal nerve block and an ultrasound-guided maxillary nerve block with ropivacaine. No analgesics were required during the six-day hospitalization period. There were no complications from the nerve blocks such as dysphagia or upper airway obstruction. The findings from this case indicated that the ultrasound-guided selective glossopharyngeal nerve block and ultrasound-guided maxillary nerve block provided effective analgesia after tonsillectomy without complications.

## Introduction

Palatine tonsillectomy is performed in patients with chronic tonsillitis or obstructive sleep apnea syndrome [1]. Electrosurgical techniques are commonly used to prevent postoperative bleeding; however, they can exacerbate postoperative inflammation, leading to increased postoperative pain [2]. This pain may affect a patient’s oral intake, return to daily activities, hospital discharge, and overall satisfaction with the procedure [3]. Multimodal approaches to managing acute postoperative pain following otolaryngology surgery usually involve combining acetaminophen, nonsteroidal anti-inflammatory drugs (NSAIDs), opioid agonists, and local anesthesia. NSAIDs and opioids can be associated with several complications [4,5]. Owing to the palatine tonsils and surrounding area receiving sensory input from the glossopharyngeal and maxillary nerves, a glossopharyngeal nerve block (GNB) is expected to reduce the pain associated with palatine tonsillectomy [6]. Although some studies have demonstrated its effectiveness, a previous report indicated that the use of postoperative analgesics remains unchanged even after GNB [7]. Therefore, more reliable nerve block methods are required. In recent years, ultrasound-guided maxillary nerve block (UGMNB) has emerged as an effective analgesic method for maxillofacial surgery. Additionally, ultrasound-guided selective GNB (UGSGNB) has been developed as a regional anesthesia method for the pharynx.

Herein, we report the case of a patient who underwent UGSGNB and UGMNB for postoperative analgesia after bilateral palatine tonsillectomy.

## Case presentation

A woman in her 30s (height, 150.9 cm; weight, 56.8 kg) visited our clinic to receive surgical consultation for recurrent acute tonsillitis occurring more than five times annually. There was no history of peritonsillar abscess or prior surgeries in the head and neck regions. A physical examination revealed bilateral palatine tonsillar hypertrophy, and both sides were classified as Grade II according to the Mackenzie classification. The patient was scheduled to undergo surgery under general anesthesia. Significant postoperative pain was anticipated; thus, UGSGNB and UGMNB were selected for effective pain relief. These nerve blocks were administered after general anesthesia induction.

**Figure 1:**
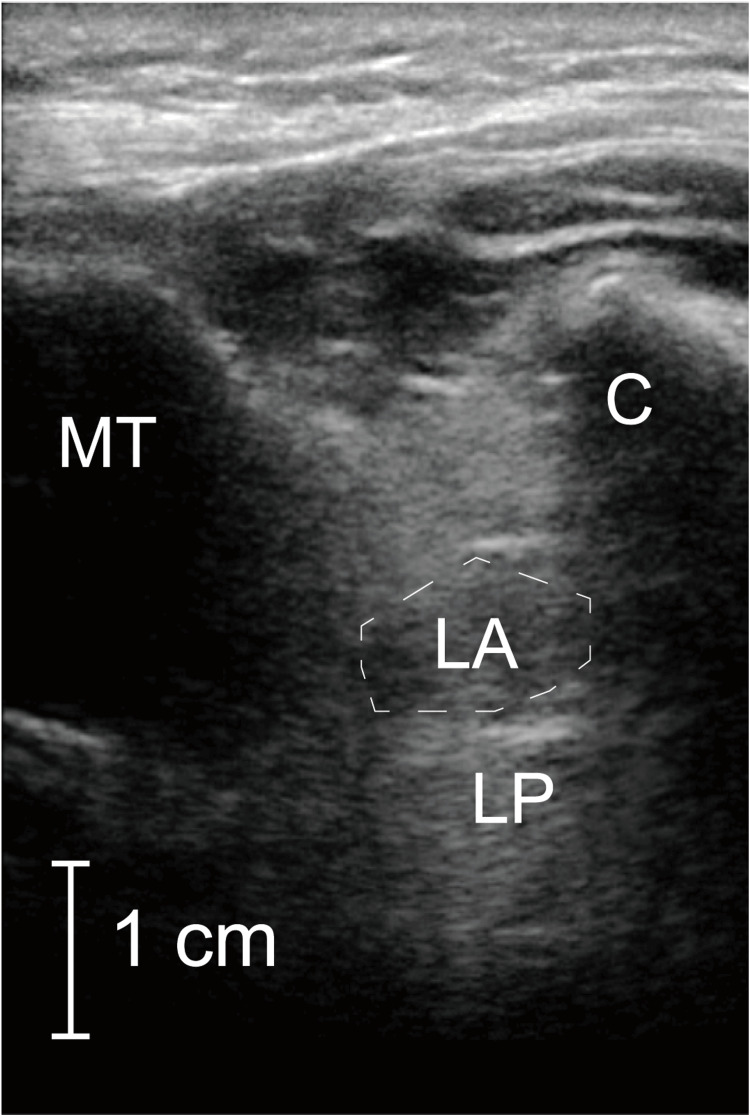
Ultrasound images of an ultrasound-guided maxillary nerve block Local anesthesia is injected after the block needle tip hits the lateral pterygoid plate. C, coronoid; LA, local anesthetic; LP, lateral plate of the pterygoid process; MT, maxillary tuberosity.

**Figure 2:**
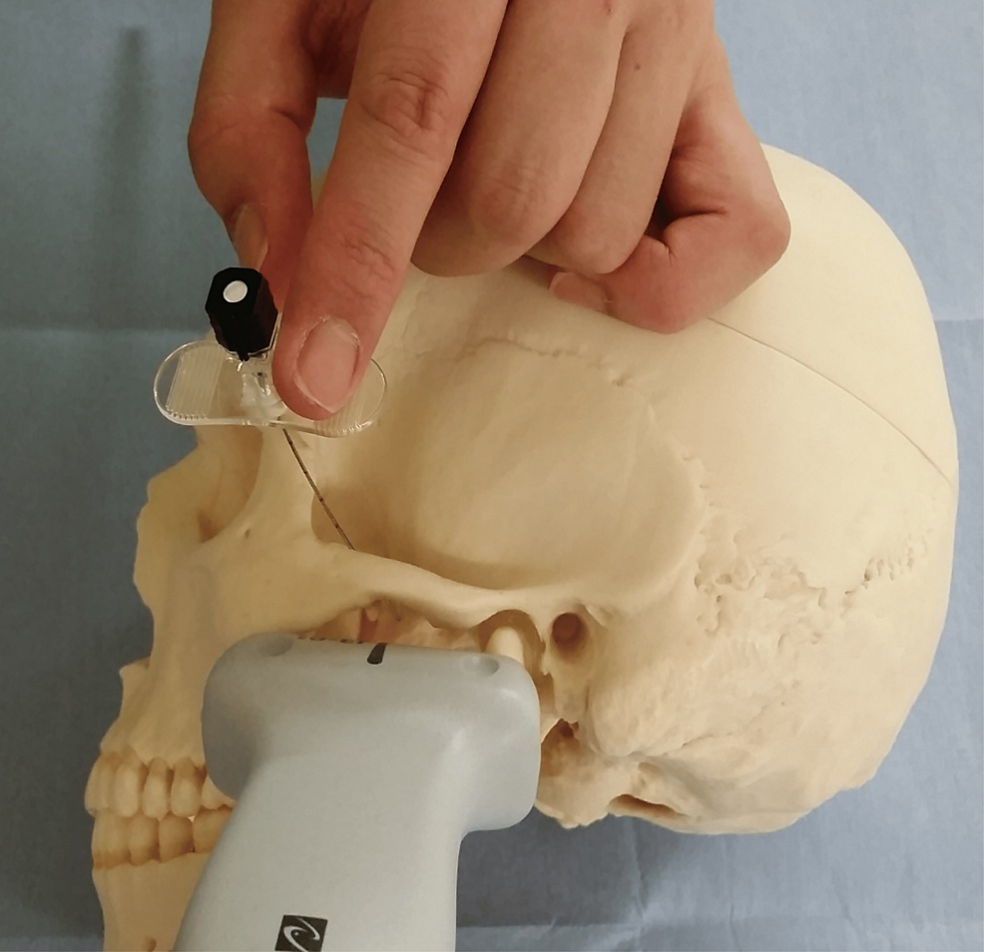
Location of the needle and ultrasound-guided equipment during ultrasound-guided maxillary nerve block A linear ultrasound probe is positioned on the face to visualize the target area. The physician should carefully examine the surrounding anatomical structures (the lateral pterygoid plate) to ensure accurate needle placement.

**Figure 3:**
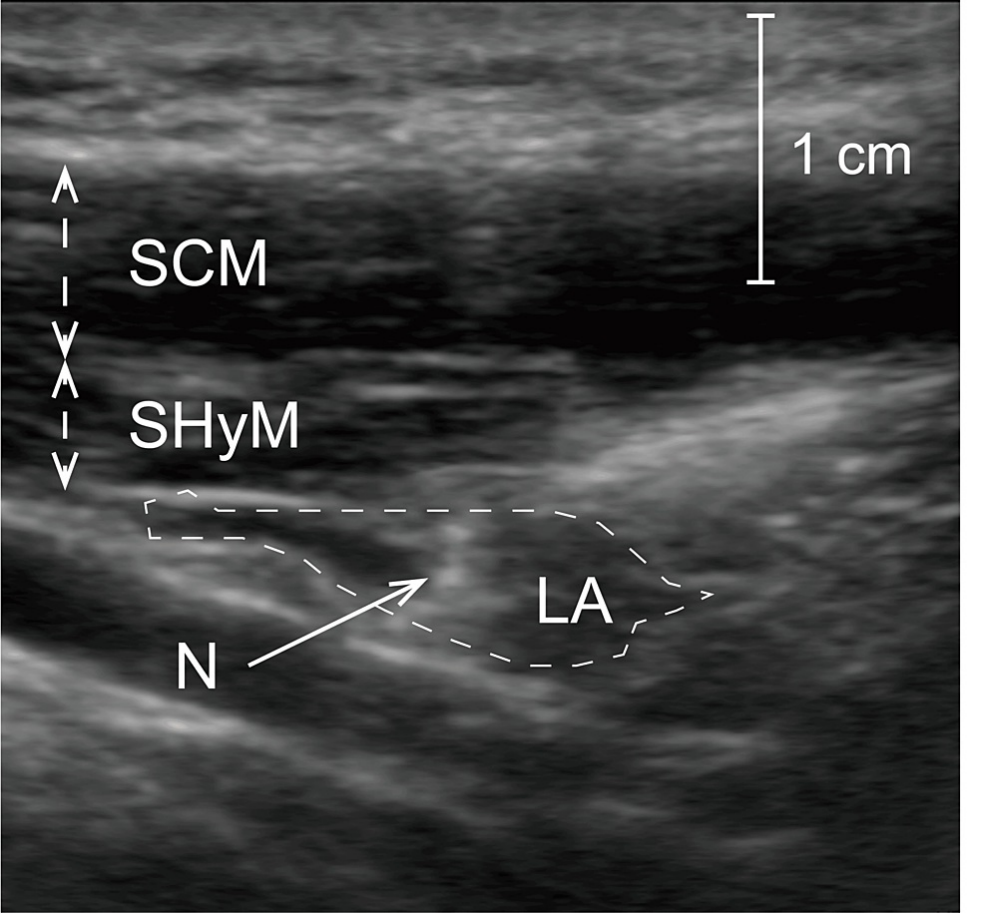
Ultrasound images of an ultrasound-guided selective glossopharyngeal nerve block The sternocleidomastoid muscle and stylohyoid muscle can be observed. We confirmed the spread of the local anesthetic. LA, local anesthetic; N, needle; SCM, sternocleidomastoid muscle; SHyM, stylohyoid muscle

**Figure 4:**
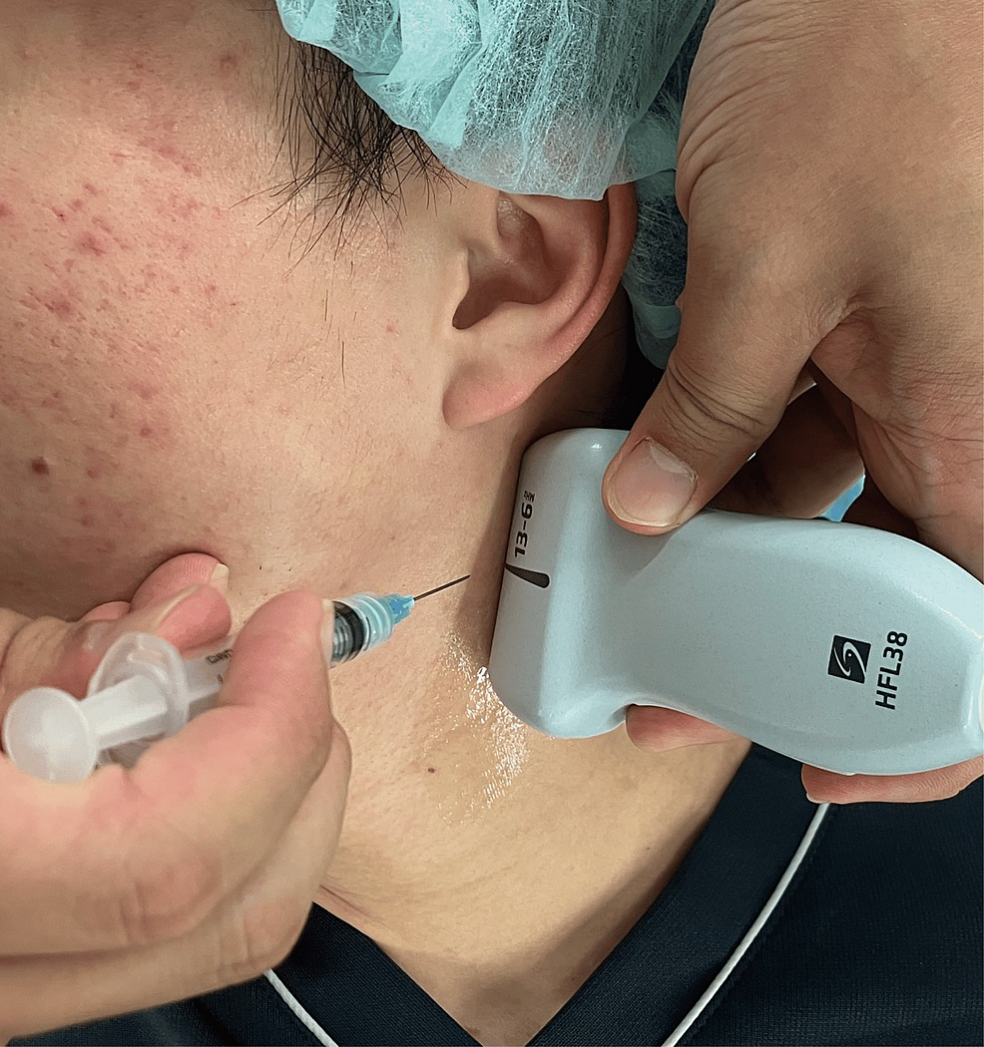
Location of the needle and ultrasound-guided equipment during an ultrasound-guided selective glossopharyngeal nerve block A linear ultrasound probe is placed on the neck to visualize the target area. The physician should meticulously assess the surrounding structures (the sternocleidomastoid and stylohyoid muscles) to ensure precise needle placement.

On arrival in the operating room, the patient underwent standard anesthetic monitoring, including pulse oximetry, sphygmomanometer measurements, capnography, and electrocardiography. After obtaining intravenous (IV) access with a 20-gauge peripheral IV catheter, general anesthesia was induced with an IV bolus of fentanyl (100 mcg) and propofol (100 mg) and maintained with desflurane (4%) and oxygen/air (1/1%; 2 L/min). Muscle relaxation was achieved with rocuronium (50 mg). A continuous infusion of remifentanil (0.2-0.25 mcg/kg/min) was administered from induction until intubation. The patient was mask-ventilated and orally intubated with a 7-mm endotracheal tube. After securing the airway, bilateral UGMNB was performed using 14 mL of plain 0.375% ropivacaine (52.5 mg) administered to the lateral pterygoid plate via a supra-zygomatic approach under ultrasound guidance (SonoSite SII; Fujifilm, Tokyo, Japan) (Figures 1, 2). Bilateral UGSGNB was performed using 4 mL of 0.2% plain ropivacaine (8 mg) administered under the stylohyoid muscle (Figures 3, 4). The patient’s heart rate and blood pressure remained stable (±20% of the baseline values) during surgery.

The surgery was completed within 51 minutes, blood loss was minimal, and the patient was extubated without challenges while spontaneously breathing and fully awake. The initial intraoperative observation of the palatine tonsils indicated that portions buried beneath the mucosa were considerably hypertrophied compared with the protruding portions within the oropharyngeal cavity. Consequently, the resection surface area was enlarged, prompting a broader scope for electrosurgical cauterization.

The immediate postoperative course was uneventful, with no episodes of delirium, postoperative nausea, or vomiting. Analgesics and antiemetics were not administered perioperatively or postoperatively because the patient experienced no pain at the surgical site. Therefore, she required no additional rescue analgesics until discharge on postoperative day 6 and had no postoperative complications. At three hours postoperatively, her numerical rating scale (NRS) score for oropharyngeal pain was 0 points, with no signs of dysphagia, upper airway obstruction, hoarseness, or stridor. Oral intake was initiated on postoperative day 1 with good food tolerance. There were no instances of re-bleeding, and as planned, the patient was discharged on postoperative day 6. No complications related to motor nerve blockage were observed.

Written informed consent was obtained from the patient to publish this case report and the accompanying images.

## Discussion

The sensory and afferent visceral nerves of the glossopharyngeal nerve primarily dominate the posterior one-third of the tongue, oropharyngeal mucosa, palatine tonsils, uvula, eustachian tube, and middle ear space [8]. Therefore, GNB is reportedly effective in managing postoperative pain following oropharyngeal surgery [9]. Conventional GNB has the risk of intravascular injection in the intraoral and extraoral approaches, which can lead to local anesthesia-induced systemic toxicity [10]. Ultrasound-guided GNB facilitates real-time visualization of the block needle, vessels, and injected local anesthetic, reducing the risk of local anesthetic intravascular injection [11].

To the best of our knowledge, this is the first report on UGSGNB as a postoperative analgesic for otolaryngology surgery. UGSGNB can be performed at a superficial depth, allowing the procedure to be conducted without damaging tissues other than muscles, reducing potential complications and patient discomfort [12]. Complications such as blocking of the sublingual and vagus nerves are side effects of GNB, potentially causing dysphagia, upper airway obstruction, hoarseness, and stridor. UGSGNB does not block the sublingual or vagus nerve [12]. The time required for bilateral UGSGNB was <5 min. The patient’s NRS score for oropharyngeal pain was 0 points throughout the hospital stay. For this patient, fentanyl was administered during anesthesia induction, and with the application of two nerve blocks, opioid-free postoperative pain was managed. The findings from this case indicated an effective analgesic influence of UGSGNB and UGMNB over an extended period, possibly because the effect of preemptive analgesia caused by these nerve blocks was large.

Future research is required to determine the extent to which analgesic effects can be obtained with UGSGNB alone. Pediatric patients have limited analgesic options compared with those available for adult patients. Therefore, the application of UGSGNB in pediatric patients should be explored. Further investigations are also required to evaluate the safety and efficacy of UGSGNB.

## Conclusions

This report demonstrates the effectiveness of UGSGNB and UGMNB as postoperative analgesia strategies for tonsillectomy. These nerve blocks provide effective extended analgesia after tonsillectomy, without complications. Our results suggest that the combination of UGSGNB and UGMNB can be an effective analgesic method in pharyngeal surgery and may be safer and simpler to perform than conventional techniques. Further investigations are required to evaluate the safety and efficacy of these nerve blocks.
